# Temporal Trends in the Impact Factor of European versus USA Biomedical Journals

**DOI:** 10.1371/journal.pone.0016300

**Published:** 2011-02-09

**Authors:** Drosos E. Karageorgopoulos, Vasiliki Lamnatou, Thalia A. Sardi, Ioannis D. Gkegkes, Matthew E. Falagas

**Affiliations:** 1 Alfa Institute of Biomedical Sciences (AIBS), Athens, Greece; 2 Department of Medicine, Henry Dunant Hospital, Athens, Greece; 3 Department of Medicine, Tufts University School of Medicine, Boston, Massachusetts, United States of America; University of Modena and Reggio Emilia, Italy

## Abstract

**Background:**

The impact factors of biomedical journals tend to rise over time. We sought to assess the trend in the impact factor, during the past decade, of journals published on behalf of United States (US) and European scientific societies, in four select biomedical subject categories (*Biology*, *Cell Biology*, *Critical Care Medicine*, and *Infectious Diseases*).

**Methods:**

We identified all journals included in the above-mentioned subject categories of Thomson Reuters Journal Citation Reports® for the years 1999, 2002, 2005, and 2008. We selected those that were published on behalf of US or European scientific societies, as documented in journal websites.

**Results:**

We included 167 journals (35 in the subject category of *Biology*, 79 in *Cell Biology*, 27 in *Critical Care Medicine*, and 26 in *Infectious Diseases*). Between 1999 and 2008, the percentage increase in the impact factor of the European journals was higher than for the US journals (73.7±110.0% compared with 39.7±70.0%, p = 0.049). Regarding specific subject categories, the percentage change in the factor of the European journals tended to be higher than the respective US journals for *Cell Biology* (61.7% versus 16.3%), *Critical Care Medicine* (212.4% versus 65.4%), *Infectious Diseases* (88.3% versus 48.7%), whereas the opposite was observed for journals in *Biology* (41.0% versus 62.5%).

**Conclusion:**

Journals published on behalf of European scientific societies, in select biomedical fields, may tend to close the “gap” in impact factor compared with those of US societies.

**What's Already Known About This Topic?:**

The impact factors of biomedical journals tend to rise through years. The leading positions in productivity in biomedical research are held by developed countries, including those from North America and Western Europe.

**What Does This Article Add?:**

The journals from European biomedical scientific societies tended, over the past decade, to increase their impact factor more than the respective US journals.

## Introduction

The most commonly used indicator of the quality or at least the popularity of scientific journals is the journal impact factor, which is not however exempt of some limitations [1], [2]. Over the past years, the impact factor of biomedical journals has generally shown a tendency to rise, which can in part be attributed to the expansion in the size of the relevant literature [3], [4]. It is well recognized also that the leading positions in productivity in biomedical research are held by developed countries, including those from North America and Western Europe [5], [6].

In this context, we sought to evaluate the trends in the impact factor of journals published on behalf of United States (US) and European biomedical scientific societies during the past 10 years.

## Methods

We did a retrospective analysis of journal impact factors provided by Thomson Reuters Journal Citation Reports® for the last 10-year period (1999–2008). We focused on 2 biomedical scientific fields, Biology and Medicine and selected two subject categories (as classified in the above-mentioned database) from each field. Specifically, we selected *Biology, Cell Biology, Critical Care Medicine,* and *Infectious Diseases.* These subject categories were chosen among those with a high median category impact factor for each field.

We retrieved all journals by name and International Standard Serial Number (ISSN) that were indexed in the above 4 subject categories of Journal Citation Reports,® for the years 1999, 2002, 2005, and 2008. To identify the journals that were published on behalf of scientific societies or professional organizations we searched in the official website of each of the retrieved journals for relevant information. We selected for inclusion the journals representing US or European scientific societies; the European countries of interest were specifically the first 15 ones to participate in the European Union (EU-15). Journals representing international scientific societies were excluded.

We extracted the impact factors of each of the included journals for the years 1999, 2002, 2005, and 2008. We grouped these data into separate variables for the US and European journals, respectively. We calculated the median value for the journal impact factor in each category and plotted graphically the temporal trends for the studied period. Journals that had an impact factor for any one of the above-mentioned years were included in this analysis.

We additionally calculated for each of the included journals the percentage change in the impact factor between 2002 and 1999, 2005 and 2002, 2008 and 2005, and finally between 2008 and 1999. Only journals that had an impact factor for both years in regard could be included in this analysis. We grouped these data into separate variables for the US and European journals and the years in regard. We assessed for statistical differences between the above variables. Our primary comparison was the difference in the percentage change in the impact factor between the US and European journals from 1999 to 2009. We used the independent samples t-test statistic for this comparison; a p-value less than 0.05 was considered as statistically significant.

## Results

The 4 subject categories of our interest *(Biology, Cell Biology, Critical Care Medicine, and Infectious Diseases)* in the Journal Citation Reports® database, included in total 252, 280, 288, and 303 journals for each of the years 1999, 2002, 2005, and 2008, respectively. Among these, we identified 167 different journals that were published on behalf of US or EU biomedical scientific societies and had an impact factor for at least one of the above years. In Table 1, we present the distribution of the journals included in our analysis with regard to region and subject category.

**Table 1:** Distribution of the journals included in our analysis according to region and subject category.

Subject category	Region		
	*US*	*EU*	*Total*
Biology	16	19	***35***
Cell Biology	40	39	***79***
Critical Care Medicine	19	8	***27***
Infectious Diseases	8	18	***26***
***Total***	***83***	***84***	***167***

Abbreviations: EU: European Union; US: United States.

**Figure 1:**
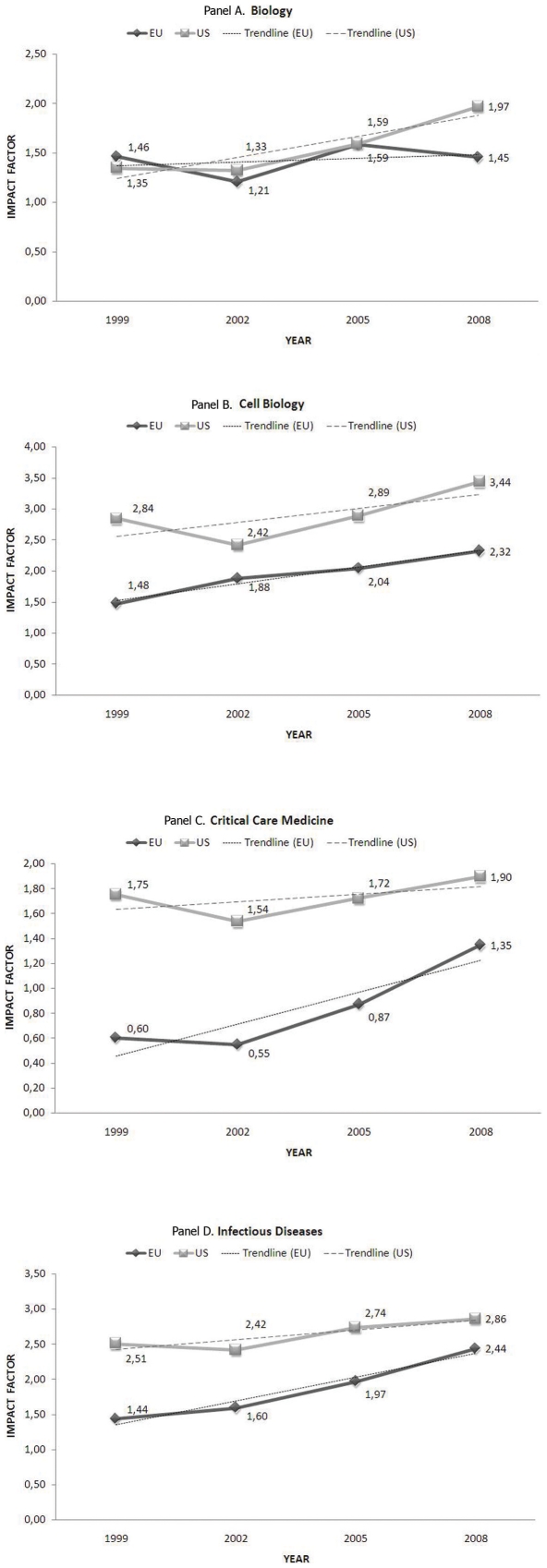
Temporal trends of the median impact factor of journals endorsed by United States (US) and European (EU) professional societies in 4 scientific categories (Panels: A. Biology, B. Cell Biology, C. Critical Care Medicine, D. Infectious Diseases). The fitted line represents the temporal trend in the median impact factors.

In Figure 1, we present graphically the temporal trends in the median impact factors of the journals included in each of the above 4 subject categories and published on behalf of US compared with EU scientific societies, in the 4 selected years between 1999 and 2008. As can be inferred from the graphs included in Figure 1, in 1998 US journals had clearly higher median impact factor compared with the European ones in the all examined subject categories, except for *Biology.* However, there appears to be a trend towards a greater increase in the median impact factor of journals published on behalf of European scientific societies compared with the US ones, in the subject categories of *Critical Care Medicine* and *Infectious Diseases*, over the past decade. The increase in the median impact factor of journals published on behalf of European scientific societies parallels that of the US ones, in the subject category of *Cell Biology*, whereas there is a trend for a greater increase in the median impact factor of journals published on behalf of the US societies, compared with the European ones, in the subject category of *Biology*.

In Table 2, we present the findings of our analysis regarding the percentage change in the impact factor of journals published on behalf of US and European scientific societies, cumulatively for all the 4 selected subject categories, between 1999 and 2008, as well as for the interval periods. Specifically, between 1999 and 2008, the impact factor of journals published on behalf of European scientific societies rose more than those published on behalf of the US ones (by 73.7±110.0%, as compared with 39.7±70.0%, p = 0.049). No relevant statistically significant difference was observed in any of the interval periods. With regard to each selected subject category, the impact factor of journals published on behalf of European scientific societies compared with US ones, rose, between 1999 and 2008, by 212.4±291.5% compared with 65.4±45.5% (p = 0.107) for *Critical Care Medicine*, 88.3±99.5% compared with 48.7±61.3% (p = 0.39) for *Infectious Diseases*, 61.7±82.0% compared with 16.3±68.7% (p = 0.029) for *Cell Biology*, and by 41.0±47.2% compared with 62.5±81.7% (p = 0. 43) for *Biology* (data not in table).

**Table 2:** Comparison of the change in impact factor of journals in 4 biomedical subject categories published by European vs US biomedical societies.

Period/Region	Number of journals	Percentage change in the IF (mean ± SD)	P-value for IF change (US vs EU)
**1999 to 2002**			
US	65	7.2±42.9%	0.09
EU	61	22.9±60.1%	
**2002 to 2005**			
US	71	12.0±31.8%	0.06
EU	68	24.4±44.7%	
**2005 to 2008**			
US	70	32.1±79.5%	0.36
EU	69	22.2±44.7%	
**1999 to 2008**			
US	59	39.7±70.0%	**0.049**
EU	56	73.7±110.0%	

Abbreviations: IF: impact factor; EU: European Union; SD: standard deviation; US: United States.

## Discussion

The main finding of our study is that the impact factor of journals published by European scientific societies rose more than that of journals published by US ones, in cumulatively 4 select subject categories, in the fields of Medicine or Biology, over the past decade. However, considerable variability was observed in this regard between journals in specific subject categories; the difference in the rise of the impact factor between European and US journals was particularly seen in the medical subject categories (*Critical Care Medicine* and Infectious Diseases) and *Cell Biology,* whereas the opposite was observed for *Biology*.

The difference in favor of the European journals regarding the degree of change in the impact factor appeared less pronounced when the median impact factor of each subject category was analyzed than when the percentage change in the impact factor of the journals included in each category was analyzed. In the first analysis, all journals that had an impact factor for at least one of the studied years were included, in contrast with the second, in which only journals that had an impact factor for both the initial and the final year compared were included. The latter analysis plausibly refers to journals with substantial tradition and influence; those with a low and declining impact factor could instead have been left out of the Journal Citation Reports® database.

A potential explanation for the relatively high increase in the impact factor of European Journals could lie in the role of the funding for research. Over the past decade, the European Union has given greater value than before on research funding, by allocating more financial resources, organizing better the process of the allocation of resources, and favoring scientific collaboration within the European Union [7]. On the other hand, the rate of research funding in the US appears to have slowed down during the past decade compared with the previous one [8]. The above differences could have resulted in a greater rise in the research productivity of Europe compared with the US [9], and this could be reflected in our study findings.

There are various factors, however, that can influence the impact factor of scientific journals [1], [10]. One of these, is the language of publication of the journals; the dominance of English in biomedical publications has well been consolidated [11]. It is possible, therefore, that editorial committees of European journals published in local languages have been tempted over the last years to change the language of publication into English, so as to increase the penetration of the journals in the global scientific community [12]. The latter could have led into a rise of their impact factor. European journals could also have achieved greater visibility among scientists through the development or advancement of electronic scientific databases, such as Scopus, that cover a greater proportion of European journals than PubMed [13].

Our study findings may be of interest to the individual researchers for deciding to what journal they should submit their work for publication. There is an important time lag between article submission, publication, and assignment of the journal impact factors for the specific year [14]. Thus, knowing the temporal trends of the impact factors in specific categories of journals, may be useful in this regard.

Our study has several potential limitations. First, it is limited to journals of 4 subject categories, two in Biology and two in Medicine. Although the biological subject categories evaluated can be considered as potentially representative of the whole field, it might not be so for the medical ones. Moreover, there was difference in our study findings between the subject categories evaluated. Other studies have also shown that the scientific contribution of researchers from different countries can differ by the discipline examined [5], [9].

We also limited our analysis to journals published on behalf of scientific societies. This has inevitably decreased the sample size we analyzed. Yet, we elected to include only the above-mentioned type of journals, as the identity of other types of journals with regard to geographical origin can be more difficult to establish with accuracy. Some bibliographic databases include such information, which is easy to retrieve, but it mostly reflects the country that the publisher of the journal is legally based in, rather than the origin of the scientific board of the journal. The journals published on behalf of scientific societies can, to our view, reflect more accurately the research productivity in each region.

Finally, our findings should be interpreted in the context of the well-discussed limitations of the journal impact factor as an indicator of the quality of scientific journals. Several scientists consider that this indicator could primarily reflect journal popularity rather than quality.[15] Moreover, there are certain ways through which journal editors can ‘manipulate’ the impact factor.[10] Still, although other relevant indicators have been developed, none has taken at least thus far the place of the journal impact factor.[2], [15]

In conclusion, our study indicates that the journals that were published on behalf of European scientific societies in 4 select biomedical subject categories tended, over the past decade, to increase their impact factor more than the respective US journals. This finding varied considerably between the 4 subject categories examined. Our analysis cannot however reflect the entire scientific fields of Biology and Medicine. Our findings could be interpreted as potentially indicative of an effort of the European Union to close the ‘gap’ in research productivity with the US.
